# Immune-Mediated Autonomic Neuropathies following Allogeneic Stem Cell Transplantation in Acute Myeloid Leukemia

**DOI:** 10.1155/2017/6803804

**Published:** 2017-08-27

**Authors:** Abhishek Mangaonkar, Hassan Al Khateeb, Narjust Duma, Erik K. St. Louis, Andrew McKeon, Mrinal Patnaik, William Hogan, Mark Litzow, Taxiarchis Kourelis

**Affiliations:** ^1^Division of Hematology, Department of Medicine, Mayo Clinic, Rochester, MN, USA; ^2^Department of Neurology, Mayo Clinic, Rochester, MN, USA

## Abstract

**Background/Aims:**

Autonomic dysfunction (AD) after allogeneic stem cell transplant (SCT) is a rare occurrence and likely immune-mediated in etiology. There is limited literature on this topic and hence, we wish to briefly describe management of two cases at our institution and their outcomes.

**Methods:**

We retrospectively identified two patients with immune-mediated AD after SCT from our database. Immune-mediated AD was defined as AD secondary to an immune-mediated etiology without an alternative cause and responding to immunosuppression.

**Results:**

The first case is of a 32-year-old man with acute myeloid leukemia (AML) who underwent double umbilical cord allogeneic SCT. The second patient was a 51-year-old woman with secondary AML who underwent matched-related donor allogeneic SCT. Both underwent an extensive work-up for an underlying etiology prior to treatment with intravenous immunoglobulin (IVIG).

**Conclusions:**

AD after SCT is a rare yet significant clinical entity. A work-up of underlying etiology should be performed. IVIG is a treatment option for these patients.

## 1. Introduction

Immune-mediated neuropathies (IMN) after autologous and allogeneic stem cell transplant (SCT) are rare, with prevalence of 0.36 percent reported among 3305 SCT patients at the Mayo Clinic [1, 2], which is higher when compared to the general population and is suggestive of a causal association between SCT and IMN [3]. Cases of immune-mediated autonomic dysfunction (AD) following SCT have also been described [4]. Herein, we describe two cases of AD after SCT responding to immunosuppressive treatment and briefly discuss their clinical course and management. We retrospectively identified patients with immune-mediated AD after a SCT seen at the Mayo Clinic between January of 1998 and January of 2016. Immune-mediated AD was defined as AD presumed secondary to an immune-mediated etiology without another plausible cause and a documented clinical response to immunosuppressive treatments.

## 2. Case  1

A 32-year-old man with a history of acute myelogenous leukemia (AML) secondary to myelodysplastic syndrome underwent double umbilical cord allogeneic SCT after fludarabine (Flu), cyclophosphamide (Cy), thiotepa, and total body irradiation (TBI) conditioning 6 months after his AML diagnosis. A few weeks after his SCT, he developed severe gastroparesis requiring the placement of a feeding tube. Following this event, his tacrolimus and prednisone doses were increased due to suspicion of graft versus host disease (GVHD) of the gastrointestinal tract. Random mucosal biopsies after an upper and lower gastrointestinal endoscopy were negative for GVHD and the patient continued to require a feeding tube for nutrition. Approximately 14 months after his transplant and while tapering of immunosuppressive agents, he developed severe symptomatic orthostatic hypotension requiring hospitalization for intravenous (IV) fluids. Adrenal insufficiency was thought unlikely due to an appropriately elevated cortisol level and a lack of response to stress dose steroids. An autonomic reflex screen demonstrated severe autonomic dysfunction. Patient underwent a tilt test for 1.5 minutes, following which there was severe progressive blood pressure drop with symptom of dizziness, weakness, and inadequate heart rate response. Beat-to-beat blood pressure responses to Valsalva maneuver showed absent late phase II and phase IV overshoot and prolonged blood pressure recovery time. The quantitative axon reflex sweat test responses were reduced at all sites except forearm. Severe orthostatic symptoms persisted despite maximal doses of midodrine and fludrocortisone and cerebrospinal fluid (CSF) analysis showed elevated protein of 109 mg/dl with normal cell count, glucose, and cytology. Serum autoimmune dysautonomia evaluation which consisted of antibodies to anti-neuronal nuclear antibodies (ANNA-1), striated muscle, acetylcholine receptor (AChR muscle binding and neuronal ganglionic), neuronal K+ channel, GAD-65, and N and P/Q type calcium channel, and ganglioside was negative. Serum and urine protein electrophoresis, HIV testing, and antinuclear antibodies were also negative. Since the development of his AD coincided with tapering of his immunosuppression, an autoimmune etiology was thought as the most likely underlying mechanism. A 5-day treatment course with intravenous immunoglobulin (IVIG) at a dose of 0.4 gm/kg daily resulted in significant improvement in orthostasis and gastroparesis. Posttreatment autonomic reflex screen was not performed due to significant clinical improvement. Soon thereafter, he was able to tolerate oral intake and his functional status significantly improved. A diagnosis of seronegative autoimmune autonomic ganglionopathy was made and he was discharged with maintenance twice weekly IVIG treatments. Orthostasis symptoms recurred following nonadherence, and resumption of IVIG led to rapid clinical improvement. At the time of last follow-up, the response has been sustained for four months.

## 3. Case  2

A 51-year-old woman with AML secondary to myelodysplastic syndrome with associated myelofibrosis underwent matched-related donor allogeneic SCT after Cy/TBI conditioning at first remission. She received cyclosporine and steroids for immunosuppression. Approximately 8 months after her SCT she developed orthostatic hypotension, confirmed by an autonomic reflex screen which showed significant adrenergic failure with mild cardiovagal and probable distal postganglionic sudomotor impairment. Heart rate responses to deep breathing and Valsalva maneuver were reduced. The quantitative sudomotor axon reflex tests were normal for all sites except marked reduction at foot. Nerve conduction studies (NCS) demonstrated reduced right ulnar and peroneal compound muscle action potential amplitudes, reduced right ulnar motor nerve conduction velocity, prolonged right peroneal motor distal latency, and borderline reduced right sural sensory nerve conduction velocity. Prolonged bilateral blink responses were also noted. Needle examination revealed scattered fibrillation potentials in both proximal and distal muscles. Mildly enlarged motor unit potentials with reduced recruitment were noted in predominantly lower extremity muscles and thoracic paraspinal muscles. Overall, NCS findings were consistent with a diffuse neurogenic disorder with demyelinating features. Antibodies to ANNA-1, amphiphysin, Purkinje Cell (PCA-1), striated muscle, N and P/Q type calcium channels, acetylcholine receptor binding, neuronal K+ channel, GAD-65, P/Q type calcium channels, and ganglioside were all undetectable. Serum protein electrophoresis did not reveal monoclonal protein. An extensive infectious diseases work-up including HIV and hepatitis panel was also negative. CSF analysis showed an elevated protein at 93 mg/dl, with otherwise normal parameters. Fludrocortisone and compression stockings were initiated, and she was treated with a five-day course of IVIG at 0.4 gm/kg daily and then switched to daily plasmapheresis and prednisone at 1 mg/kg. Plasmapheresis was slowly tapered off to three and then 2 times per week as symptoms improved. Steroids were tapered over 2 weeks. She responded to this therapy within 2 weeks and eventually orthostatic symptoms resolved by the end of the hospitalization. A posttreatment autonomic reflex screen showed normal heart rate responses to deep breathing and Valsalva maneuver and normal quantitative sudomotor axon reflex tests at all sites except the foot. Her leukemia eventually relapsed after four months and she passed away subsequently from an unrelated cause.  Table 1 summarizes the results of clinical, laboratory tests, treatment, and its response in the two cases.

## 4. Discussion

In this brief report, we demonstrate that autonomic IMNs may rarely develop late after an allogeneic SCT. They are associated with significant morbidity but fortunately tend to respond rapidly to treatments directed at the antibody immune response, such as IVIG and plasmapheresis.

Demyelinating neuropathies following SCT have been identified in 0.5% patients [4]. IMNs with isolated autonomic dysfunction are even rarer and limited to isolated case reports [1, 4, 5]. Nakane et al. found that all 5 patients undergoing a reduced intensity SCT developed a decrease in heart rate variability, indicative of autonomic dysfunction [6].

The possible underlying pathophysiologic mechanisms for IMNs following SCT are an immune reconstitution syndrome, GVHD, or a paraneoplastic phenomenon [1]. In both our cases, disease relapse was ruled out and response to immunosuppression was rapid and sustained, supportive of an immune-mediated mechanism. Furthermore, the absence of skin, GI, or liver involvement made GVHD appear less likely. However, neurologic manifestations of chronic GVHD can also present as IMNs [7]. Acute or chronic inflammatory demyelinating polyneuropathies are two examples of transplant-related IMNs impacting large fiber nerves [8]. These immune-mediated neurologic disorders seem more common in allogeneic SCT patients when compared to the general population, which suggests a causal association [3]. Karam et al. demonstrated that such disorders are also seen following autologous SCT, suggesting that the underlying pathogenesis may be related to a nonspecific activation of the host's immune system rather than a graft versus host mechanism [1]. Further, the fact that both patients had an elevated CSF protein is supportive of an inflammatory process.

An inferred autoimmune etiopathologic mechanism is important to guide empiric therapy. In our two patients, favorable response to IVIG and plasmapheresis is specifically supportive of an antibody-mediated mechanism. Long term outcomes were favorable for both patients consistent with previous reports, although clinical improvement can be incomplete with persistent residual deficits [9, 10].

Limitations of our study include its retrospective nature, limited number of patients, and the short follow-up. However, we corroborate prior results that IMN following SCT is rare and a thorough work-up for relapsed disease and other causes of AD (chemotherapy, infectious, paraneoplastic, and endocrinologic etiologies) is critical prior to initiating empiric therapy for a presumed autoimmune cause. Furthermore, we probably underestimated the incidence of immune-mediated autonomic neuropathy due to lack of uniform diagnostic criteria and some patients may have limited autonomic disorders, such as isolated gastrointestinal dysmotility that may be poorly recognized. Our limited experience suggests that an empiric short-term trial of IVIG or plasma exchange is reasonable, with continued maintenance treatment for patients who exhibit a therapeutic response. Additional therapies directed at reducing antibodies against self-antigens such as rituximab and plasmapheresis may be reasonable options for long term management of these patients. Steroids may also be beneficial, at least in the short-term. Further analysis of a larger patient cohort will be necessary to confirm the association of IMN with SCT and potentially identify clinical factors that may predict IMN occurrence, prognosis, and best therapeutic approaches.

## Figures and Tables

**Table 1:** Table summarizing results of clinical/laboratory testing, treatment response, and outcomes in the two cases.

Test	Case 1	Case 2
Autonomic reflex screen	(i) Moderate cardiovagal, widespread postganglionic sudomotor and severe adrenergic impairment on this study. (ii) Decreased heart rate responses to deep breathing and Valsalva maneuver. (iii) QSWEAT responses reduced at all sites except the forearm.	(i) Significant adrenergic failure with mild cardiovagal and probable distal postganglionic sudomotor impairment. (ii) Heart rate responses to deep breathing and Valsalva maneuver were reduced. (iii) The quantitative sudomotor axon reflex tests were normal for all sites except marked reduction at foot.

Antibody tests (all were undetectable) ^*∗*^Antibodies tested in addition for case 2	ANNA-1, striated muscle, acetylcholine receptor (AChR muscle binding and neuronal ganglionic), neuronal K+ channel, GAD-65, and N- and P/Q type calcium channel, ganglioside, amphiphysin^*∗*^, Purkinje Cell (PCA-1)^*∗*^.	

Treatment	IVIG 0.4 gm/kg for 5 days.	IVIG at 0.4 gm/kg for 5 days, daily plasmapheresis, prednisone at 1 mg/kg.

Response	Significant symptomatic improvement. Posttreatment autonomic reflex testing not performed.	Significant symptomatic improvement in two weeks. Posttreatment autonomic reflex screen showed normal heart rate responses to deep breathing and Valsalva maneuver and normal quantitative sudomotor axon reflex tests at all sites except the foot.

## References

[1] Karam C., Mauermann M. L., Johnston P. B., Lahoria R., Engelstad J. K., Dyck P. J. B. (2014). Immune-mediated neuropathies following stem cell transplantation. *Journal of Neurology, Neurosurgery and Psychiatry*.

[2] Kang J.-M., Kim Y.-J., Kim J. Y. (2015). Neurologic complications after allogeneic hematopoietic stem cell transplantation in children: analysis of prognostic factors. *Biology of Blood and Marrow Transplantation*.

[3] Lehmann H. C., Horste G. M. Z., Hartung H.-P., Kieseier B. C. (2009). Pathogenesis and treatment of immune-mediated neuropathies. *Therapeutic Advances in Neurological Disorders*.

[4] Delios A. M., Rosenblum M., Jakubowski A. A., Deangelis L. M. (2012). Central and peripheral nervous system immune mediated demyelinating disease after allogeneic hemopoietic stem cell transplantation for hematologic disease. *Journal of Neuro-Oncology*.

[5] Roskrow M. A., Kelsey S. M., McCarthy M., Newland A. C., Monson J. P. (1992). Selective automatic neuropathy as a novel complication of BMT. *Bone Marrow Transplantation*.

[6] Nakane T., Nakamae H., Muro T. (2009). Cardiac and autonomic nerve function after reduced-intensity stem cell transplantation for hematologic malignancy in patients with pre-transplant cardiac dysfunction. *Annals of Hematology*.

[7] Grauer O., Wolff D., Bertz H. (2010). Neurological manifestations of chronic graft-versus-host disease after allogeneic haematopoietic stem cell transplantation: report from the consensus conference on clinical practice in chronic graft-versus-host disease. *Brain*.

[8] Suzuki S., Mori T., Mihara A. (2007). Immune-mediated motor polyneuropathy after hematopoietic stem cell transplantation. *Bone Marrow Transplantation*.

[9] Koike H., Watanabe H., Sobue G. (2013). The spectrum of immune-mediated autonomic neuropathies: insights from the clinicopathological features. *Journal of Neurology, Neurosurgery and Psychiatry*.

[10] Koike H., Atsuta N., Adachi H. (2010). Clinicopathological features of acute autonomic and sensory neuropathy. *Brain*.

